# Unilateral Aplasia versus Bilateral Aplasia of the Vertebral Artery: A Review of Associated Abnormalities

**DOI:** 10.1155/2017/7238672

**Published:** 2017-08-28

**Authors:** L. Vasović, M. Trandafilović, S. Vlajković, G. Djordjević, M. Daković-Bjelaković, M. Pavlović

**Affiliations:** ^1^Department of Anatomy, Faculty of Medicine, University of Niš, 81 Blvd. Dr. Zoran Djindjić, 18000 Niš, Serbia; ^2^Health Center Niš, 15 Vojvode Tankosića St., 18000 Niš, Serbia

## Abstract

Morphological characteristics of 108 cases of uni- and bilateral aplasia of the vertebral artery (VA) in reports or images of retrospective studies, including one recent case, published between 1967 and 2016 are analyzed. Incidence, gender, persistence of carotid-vertebrobasilar anastomosis (CVBA), associated with other vascular variants, and vascular pathology in each group of uni- and bilateral VA aplasia are mutually compared. Most of the cases of VA aplasia in ages 31 to 80 were discovered in USA, Japan, and India. The bilateral VA aplasia is more common in the male gender than in the female one. The side of the VA aplasia had a significant effect on the side of CVBA persistence. Associated aplasia of other arteries was more common in cases of unilateral VA aplasia. The left VA was more commonly hypoplastic in cases of single right VA aplasia than the right VA in cases of single left VA aplasia. Aneurysms of definitive arteries were more frequent in cases of single right VA aplasia than in cases of single left VA aplasia. We claim that the aplasia of the VA probably depends on genetic factors in some races, while diseases are expressed usually in persons over 30 years of age.

## 1. Introduction

The development of paired vertebral arteries (VAs) of 7 mm to 12 mm of the human embryo provides longitudinal anastomoses of so-called primitive proatlantal intersegmental artery (PIA) and six cervical intersegmental arteries (CIAs) that arise from the dorsal aorta on both sides. Actually, the sixth CIA according to Padget [1], or the seventh CIA according to Effmann et al. [2], becomes the VA and contributes to the subclavian artery (SA) in this embryonic stage, while the primitive PIA also supplies a caudal part of precursors of the basilar artery (BA), that is, paired longitudinal neural arteries (LNAs) on the developing hindbrain. After this period, the VA from its origin courses through prevertebral (V1), cervical (V2), atlantic (V3), and intracranial (V4) topographical parts before its connection with opposite artery in the BA [3].

The development of the internal carotid artery (ICA) is independent of the VA. Namely, only one part of the primitive ICA derives from the third primitive aortic arch, while all other ICA segments represent cranial extensions of the dorsal aorta on both sides. Transitory vascular channels or primitive carotid-vertebrobasilar anastomoses (CVBAs) between the LNAs and ICAs exist at a time when embryonic length is 4-5 mm [4–8]. The primitive hypoglossal (PHA), primitive otic (POA), and primitive trigeminal (PTA) arteries are determined, as cited [5–8], by their relationship with hypoglossal nerve, otic vesicle, and trigeminal ganglion, respectively, while the PIA is named according to the course between the occipital and cervical somites [1, 6]. With the formation of the posterior communicating artery (PCoA) cranially and vertebrobasilar system caudally, CVBAs regress and usually disappear by the 14 mm stage of human embryo.

Doppler sonography of health infants showed that 114/7991 (1.4%) infants had unilateral VA aplasia—left in 0.51% and right in 0.91% of cases [9]. The persistence of CVBA(s) in cases of uni- or bilateral aplasia of the VA or in cases of normal VA on both sides after this period is conditioned by different vascular factors and however insufficiently explained [5–8, 10].

Recent finding of aplasia of the right VA followed by the persistence of the left PTA and so-called intermediate communicating artery on the right side inspired the authors to review literature cases with established uni- and bilateral VA aplasia and their relationships with persistence of primitive or definitive anastomoses and/or vascular variants.

## 2. Methods

Morphological characteristics of 108 cases of total uni- and bilateral aplasia of the VA in single reports or images of retrospective studies published between 1967 and 2016 are separately analyzed. We included one personal case, as well as literature cases in articles available on Google display network or in the library archive of our Faculty of Medicine. Five general parameters, incidence, gender, persistence of CVBA, other associated vascular variants, and associated vascular pathology, in each group of uni- and bilateral VA aplasia are mutually compared.

So-called intermediate communicating artery (ICoA) is defined according to its schematic presentation in the book of Microneurosurgery [11] and previous findings in the fetuses [12] and adult cadavers [13].

### 2.1. Patient Population

31/108 (28.70%) cases of bilateral VA aplasia, 46/108 (42.59%) cases of the left VA aplasia, and 31/108 (28.70%) cases of the right VA aplasia including recent one were selected. All cases belonging to different populations, gender, and age in appropriate tables are classified (Tables 1–3).

### 2.2. Statistical Analysis

The incidences of all cases of uni- and bilateral VA aplasia in appropriate tables were noted; statistical test, *χ*^2^ nonparametric test, was used. Statistical significance was noted in a case of *p* < 0.05. Statistical analysis was performed using statistical software of IBM Corp., released in 2011, and IBM SPSS Statistics for Windows, Version 20.0, IBM Corp., Armonk, NY.

## 3. Theoretical Background

### 3.1. Bilateral VA Aplasia

There are cases without data about gender and 11/31 female and 19/31 male cases of bilateral VA aplasia; age of these cases ranged from 14 days to 76 years.

Initial symptoms or reasons of discovery of 31 cases of bilateral VA aplasia were different and unspecified. Among primary symptoms, a headache in 6/31 cases [14–19], vertigo in 5/31 cases [20–24], and weakness in 4/31 cases [25–28] preceded the discovery of VA aplasia.

Although some primitive CVBA(s) persisted in 29/31 cases, there were bilateral findings for persistent PHA (PPHA) in one [29], persistent PTA (PPTA) in two [19, 27], and persistent PIA (PPIA) in eight cases [15, 19, 24, 25, 28, 30–32]; a simultaneous presence of bilateral PPIAs and left PPTA in one case was documented [19]. Unilaterally, CVBA persisted in 19 of cases (10 on the left and 8 on the right side); there was no data about the side of the PPHA in 1 out of 19 cases [33]. A persistence of CVBA was as follows: (1) PIA persisted 17 times—8 bilaterally, 5 on the left side, and 4 on the right side [14, 15, 17, 19, 20, 22–25, 28, 30–32, 34–37]; (2) PHA persisted 10 times—1 bilaterally, 5 on the left side, 3 on the right side, and 1 without data [16, 21, 26, 29, 33, 38–42]; (3) PTA persisted 3 times—1 bilaterally, 1 on the left side (associated with bilateral PPIAs), and 1 on the right side [19, 27, 43].

Two cases of associated arterial anastomoses and bilateral VAs aplasia were exceptions. Namely, Tsai et al. [18] have described that the left occipital-vertebral anastomosis enabled posterior circulation in a 36-year-old male, while Pauliukas [44] presented the BA in continuation of the left occipital artery (OA) on an angiogram.

CVBAs were only vascular variants associated with bilateral VA aplasia in 18/31 cases.

Otherwise, associated aplasia of other vessels was related to the PCoA that was found bilaterally in 3/31 cases [20, 33, 41] and unilaterally in 1 out of 31 cases [23], as well as to the BA also in one case [43], and to some dural sinuses simultaneously with bilateral internal jugular veins in another case [30].

Other associated vascular variations in 13/31 cases were found, mostly in the form of unusual side branches or fetal origin of the posterior cerebral artery (PCA) or additional anastomoses. So, the BA as a continuation of the left OA [44], the OA as a branch of the PPIA [25], or the posterior inferior cerebellar artery (PICA) as BA branch [21, 25] or PPHA branch [33] was described. Fetal origin of the PCA [23, 43] and anastomoses with thyrocervical and/or OA branches [22, 27] in two particular cases were found. Enlargement of some dural sinuses and Galen's vein malformation [30], hypoplasia of bilateral posterior communicating arteries [42], irregular caliber of ICA [14], or tortuous course of the PPHA [41] was found in single cases.

Vascular pathology was noted in 16/31 cases. Aneurysms of different arteries, BA [16, 42], anterior communicating artery [16, 17, 33], ICA [43], PPHA [41], as well as stenosis of carotid arteries [20, 23, 27, 31–33, 35, 38, 42], or cerebral infarction [18, 25, 27] among them, angiographically were confirmed.

### 3.2. Single Left VA Aplasia

There were 24/46 female cases and 20/46 male cases and no data for 2 cases of single left VA aplasia; age of these cases ranged from the stillborn to the 83-year-old ones.

Initial symptoms or reasons of discovery of 46 cases of the left VA aplasia were also different and unspecified. A headache in 10 cases [45–54], cavernous hemangioma in 7 cases [55, 56], and vertigo in 4 cases [57–60] were relatively frequently evidenced.

Primitive CVBA(s) persisted in 40/46 cases, mostly unilaterally, that is, in 35 cases on the left side, 3 cases on the right side [54, 56], and 2 cases on both sides [46, 61]. Two cases of single left VA aplasia were associated with the persistence of two CVBAs also on the left side [62, 63]. A persistence of CVBA was as follows: (1) PIA persisted 20 times on the left side [30, 45, 47, 50, 53, 57, 59, 62–73], whereby it had a common trunk with PPHA at origin in one case [62], and was associated with the left PPTA in the second case [63]; (2) PHA persisted 16 times (as a single vessel in 13 cases, bilaterally in 2 cases, and as a common trunk with PPIA in one case) [46, 48, 49, 51, 52, 55, 60–62, 74–79]; (3) PTA persisted 6 times—3 cases on the left (simultaneously with the left PPIA in one case) [56, 63, 80] and 3 cases on the right side [54, 56].

CVBAs were only vascular variants associated with the left VA aplasia in 30/46 cases.

Associated aplasia of other vessels was related to the left ICA in 3 cases [56, 81], to the PCoA in 8 cases, 4 times on the left side [47, 60, 74, 75] and bilaterally in 4 cases [48, 52, 63, 79], to the left anterior inferior cerebellar artery (associated with the left PCoA aplasia) in one case [75], to the right anterior cerebral artery in one case [65], and to the left common carotid artery (CCA) simultaneously with subclavian artery (SA) in one case [81].

Other associated vascular variations in 34/46 cases were found, mostly in the form of arterial hypoplasia or unusual origin and/or branches and/or termination. Associated hypoplasia of the right VA in 16/46 cases was documented (Table 4), whereas its hyperplasia was only in two cases [54, 56]. Associated unusual branches were in 8/46 cases [30, 48, 56, 61, 63, 73, 75], while a termination of the right VA as the PICA in 5/46 cases [47, 63, 66, 70, 79] and arteriovenous malformation in 4/46 cases [49, 51, 53, 64] were also noted. Some congenital anomalies in 7/46 patients were reasons of discovery of the left VA aplasia [30, 56, 61, 81].

Vascular pathology in 24/46 cases was noted; aneurysms of different arteries, ICA [57, 81], PCA [64], PICA [45], PPHA–PICA junction [48], PPHA-BA junction [52], ACA [52], SA [65], as well as stenosis of ICA [60, 67, 70, 78], CCA [66, 78], PPHA [78, 79], or SA [65, 69] and cerebral hemorrhagic lesions [46, 61, 65, 68, 69, 74], angiographically were confirmed.

### 3.3. Single Right VA Aplasia

There were 14/31 female cases, 14/31 male cases, and 3/31 cases without data about gender of single right VA aplasia; age of these cases ranged from 4 days to 79 years.

Initial symptoms or reasons of discovery of 31 cases of the right VA aplasia were also different and unspecified. Among primary symptoms, a headache in 5 cases [75, 82–85], weakness in 4 cases [86–89], or vertigo in 3 cases [90–92] was evidenced.

**Figure 1:**
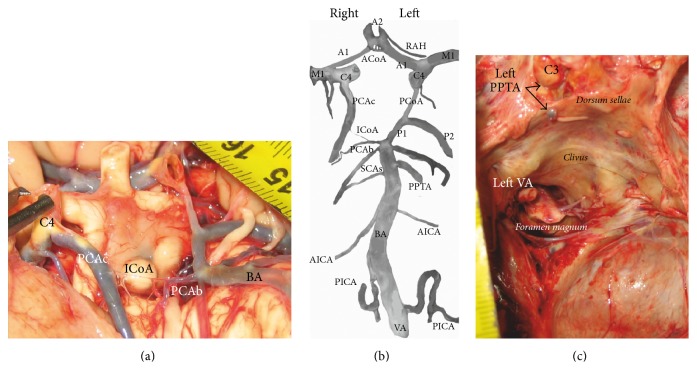
Some arteries of the carotid and vertebrobasilar systems on the brain base and in the middle and posterior cranial fossae in a 77-year-old man autopsied because of myocardial infarction in the Institute of Forensic Medicine; the approval for coauthor's (MT) investigation of cadaveric cases was obtained from the Research Ethics Committee (number 01-9068-4) of our Faculty of Medicine. (a) Additional vascular component, so-called intermediate communicating artery (ICoA) between the right posterior cerebral artery (PCA) of carotid (C4) origin (PCAc) and right PCA of basilar (BA) origin (PCAb) in the cerebral arterial circle marking, is separately shown. (b) Main arteries of the carotid and vertebrobasilar systems from original picture are extracted and marked. The cerebral arterial circle has the shape of a decagon; its vascular components are as follows: subparts of the cerebral parts (C4) of paired internal carotid arteries, precommunicating part (A1) of paired anterior cerebral arteries connected by network configuration of the anterior communicating artery (ACoA), and then the left posterior communicating artery (PCoA), which connects and divides ipsilateral PCA in the precommunicating (P1) and postcommunicating (P2) parts. So-called ICoA connecting the right PCAc and PCAb presents the tenth vascular component in the cerebral arterial circle. Additional BA branch on the left side, located below the superior cerebellar artery (SCA), as a persistent primitive trigeminal artery (PPTA) is marked. Besides Heubner's artery (RAH), a large side branch of the left anterior cerebral artery and sphenoidal part (M1) of paired middle cerebral arteries are also marked. The right SCA, as partially duplicated vessel and single left SCA, as well as single anterior inferior cerebellar artery (AICA) on both sides and the right posterior inferior cerebellar artery (PICA) are side branches of the BA, while the left PICA is a branch of ipsilateral vertebral artery (VA). (c) View on a part of the middle and posterior cranial fossae of the same case. It shows the left PPTA as a branch of the cavernous part (C3) of the internal carotid artery and only the left VA in the course through the foramen magnum.

Primitive CVBA(s) persisted in 25/31 cases, mostly unilaterally, that is, on the right side (21/25), except 4 cases of its persistence on the left. A persistence of CVBA was as follows: (1) PIA persisted 5 times—4 on the right [85, 92–94] and 1 on the left side [91]; PHA persisted 16 times—15 on the right [40, 82–84, 87, 95–104] and 1 on the left side [75]; PTA persisted in a case described by Möller-Hartmann et al. [90] and in recent case (Figure 1); unnamed right external carotid-vertebral anastomosis persisted in one case [89], “unusual” right CVBA in another case [88], and ICoA in the recent case (associated with the left PPTA).

Associated aplasia of other vessels was related to the ICA on the right side in one case [105] and on the left side in the second case [106], bilateral external carotid arteries (ECAs) in one case [92], right SA branches in one case [107], BA in one case [90], unilateral PCoA in 4 cases including recent case [75, 93, 96], and bilateral PCoAs in three cases [87, 88, 99] and the right ACA (associated with bilateral PCoA aplasia) in one case [88].

Other associated vascular variations in 23/31 cases are found, mostly in the form of arterial hypoplasia or unusual origin or course and/or branches and/or termination. Associated hypoplasia of the left VA in 13/31 cases was documented (Table 5). Variable origin of some arteries in 5/31 cases including recent one was found [88, 92, 95, 105]. Associated unusual branches or termination of arteries in 9/31 cases including the recent one [75, 83, 86, 88, 90, 92, 94, 107] was documented.

Vascular pathology in 20/31 cases was noted. Aneurysms of different arteries, middle cerebral artery [83, 94], both anterior choroidal arteries [85], VA [108], PPHA [82], BA [99], posterior inferior cerebellar artery [103], or multiple cerebral arteries [101], usually in single cases were discovered. Stenosis of ICA in 8/31 cases was found [84, 87, 90, 93, 95, 96, 104, 105]. Occlusion of some cerebral arteries was also evidenced in 5/31 cases [89, 95, 96, 99, 100].

### 3.4. Single Left VA versus Bilateral VA Aplasia

Calculated incidences of selected morphological parameters in Table 6 are presented.

1. Incidence was as follows: single left VA aplasia was more common than bilateral VA aplasia.
2. Gender was as follows: although female gender was frequent in cases of single left VA aplasia, male gender was more frequent in cases of bilateral VA aplasia; generally, there was no significant sex difference in cases of the left VA aplasia, either single or associated with the right VA aplasia.
3. Persistence of CVBA was characteristic as follows: (A) there was significant incidence of unilateral persistence of CVBA in cases of both single left and bilateral VA aplasia, especially in cases of single left VA aplasia; (B) as bilateral CVBA persisted in one-third of cases of bilateral VA aplasia, one can say that this bilateral persistence was not the rule; (C) low incidence of persistence of two different CVBAs in both single left and bilateral VA aplasia was found; (D) PIA persisted in about one-half of the cases; however, it was more common in cases of bilateral VA aplasia; (E) PHA persisted in one-third of cases of both single left and bilateral VA aplasia; and (F) PTA persisted in both single left and bilateral VA aplasia with almost equal (low) frequency.
4. Additional vascular variants were as follows: (A) aplasia of different arteries, CCA, ICA, ACA, PCoA, anterior inferior cerebellar artery [AICA], or SA, followed single left VA aplasia, while aplasia of only PCoA and BA was followed by bilateral VA aplasia; (B) absence of some dural sinuses and internal jugular veins only in one case of bilateral VA aplasia was associated; (C) one-third of cases of hypoplastic right VA with the left VA aplasia were associated; and (D) more than one-third of cases of other vascular variants, such as unusual origin or side branches or termination or additional anastomoses with single left VA and bilateral VA aplasia, were associated.
5. Associated vascular pathology was presented as follows: (A) aneurysms of different definitive cerebral arteries in one-fifth of cases of bilateral VA aplasia were found; (B) rare aneurysms of CVBAs characterized cases of single left and bilateral VA aplasia; (C) there was high incidence of other cerebral pathology (stenosis or occlusion or cerebral infarction or stroke) in cases of single left and bilateral VA aplasia; and (D) low incidence of noncerebral pathology characterized only cases of single left VA aplasia.

### 3.5. Single Right VA versus Bilateral VA Aplasia

Calculated incidences of selected morphological parameters in Table 7 are presented.

1. Incidence was as follows: right VA aplasia was more common when associated with left VA aplasia than when it was a single abnormality.
2. Gender was as follows: although female gender was frequent in cases of single right VA aplasia while male gender was more common in cases of bilateral VA aplasia, generally, male gender was more common in cases of both single right and bilateral VA aplasia.
3. Persistence of CVBA was characteristic as follows: (A) there was significant incidence of unilateral persistence of CVBA in cases of both single right and bilateral VA aplasia, especially in cases of single right VA aplasia; (B) bilateral persistence of CVBA in cases of single right VA aplasia was not found; it was significant in cases of bilateral VA aplasia; (C) there was no persistence of two different CVBAs in cases of single right VA aplasia, while they persisted with low incidence in cases of bilateral VA aplasia; (D) there was significant persistence of PIA in cases of bilateral VA aplasia in regard to single right VA aplasia; (E) there was persistence of PHA in one-half of cases of single right VA in regard to one-third of cases of bilateral VA aplasia; (F) PTA persisted in both right and bilateral VA aplasia with almost the same (low) frequency; and (G) there was, also, low frequency of persistence of additional arterial anastomoses in cases of both single right and bilateral VA aplasia.
4. Additional vascular variants were as follows: (A) aplasia of different arteries, ECA, ICA, ACA, PCoA, BA, and SA branches, characterized single right VA aplasia, while aplasia of only two arteries (BA and PCoA) was associated with bilateral VA aplasia; (B) associated unilateral PCoA aplasia was more common in cases of single right VA aplasia; (C) there were about one-half of the cases of hypoplastic left VA in cases of aplasia of the right VA; and (D) different vascular variants in one-third of cases of single right VA aplasia were associated, while they were more frequent in cases of bilateral VA aplasia.
5. Associated vascular pathology was presented as follows: (A) aneurysms of definitive arteries were more common in cases of single right VA aplasia than in cases of bilateral VA aplasia; (B) aneurysms of CVBAs were rare findings in cases of single right and bilateral VA aplasia; (C) other cerebral pathology in one-third of cases of single and bilateral VA aplasia was discovered; and (D) low incidence of noncerebral pathology in cases of single right and bilateral VA aplasia was found.

### 3.6. Single Left VA versus Right VA Aplasia

Calculated incidences of selected morphological parameters in Table 8 are presented.

1. Incidence was as follows: single left VA aplasia was more common than single right VA aplasia.
2. Gender was as follows: although female and male gender were more common in cases of left VA aplasia than in cases of right VA aplasia, there was no significant sex difference in cases of unilateral VA aplasia.
3. Persistence of CVBA was characteristic as follows: (A) incidence of persistence of CVBA was high and almost equal in both cases of unilateral VA aplasia, but not as an absolute rule; (B) CVBA always presented if one VA is aplastic and the other VA is hypoplastic; (C) there was no bilateral persistence of CVBAs in cases of single right VA aplasia, while it was rare finding in cases of single left VA aplasia; (D) there was no persistence of two different CVBAs in cases of single right VA aplasia, while it was a possible (rare) finding in cases of single left VA aplasia; (E) PIA persisted more frequently in cases of single left VA aplasia; (F) PHA persisted in one-third of cases of single left VA aplasia and in one-half of cases of single right VA aplasia; (G) although PTA persisted with low incidence in cases of single left VA aplasia, it was twice more often than on the right side; and (H) there were no additional vascular anastomoses in cases of single left VA aplasia, while they were rare findings in cases of single right VA aplasia.
4. Additional vascular variants were as follows: (A) aplasia of the same four arteries, CCA, ICA, ACA, and PCoA, for both cases was characterized; aplasia of the AICA and SA was specific for single left VA aplasia, while aplasia of BA and SA branches was specific for single right VA aplasia; (B) there were more than one-third of cases of hypoplastic VA associated with aplasia of opposite VA; (C) the left VA was more commonly hypoplastic in cases of single right VA aplasia than the right VA in cases of single left VA aplasia; (D) characteristic finding was associated aplasia of other arteries in 8/16 and 6/13 cases, respectively, of mutual aplasia of one VA and hypoplasia of the other VA; and (E) associated vascular variants (except those of a vessel's aplasia and presence of CVBA) in one-third of cases of single left or right VA aplasia were presented.
5. Associated vascular pathology was presented as follows: (A) aneurysms of definitive arteries were more frequent in cases of single right VA aplasia than in cases of single left VA aplasia; (B) aneurysms of CVBAs were rare findings in both cases of unilateral VA aplasia; (C) different cerebral pathology in cases of single left and right VA aplasia in more than one-third of cases was documented; and (D) low incidence of noncerebral pathology, especially in cases of single left VA aplasia, was noted.

### 3.7. Uni- and Bilateral VA Aplasia versus Persistence of CVBA

Generally, a relationship of the VA aplasia and persistence of CVBA was as follows: (A) the side of the VA aplasia has significant influence on the side of CVBA persistence (*p* < 0.001); (B) CVBA is significantly more common on the left side (*p* = 0.046 (*p* < 0.05)) independently of the side of VA aplasia; and (C) CVBA of ICA origin is significantly more common (*p* < 0.001).

## 4. Conclusions

Summarizing previous data, we point out the following facts:

1. Almost 50% of cases of uni- and bilateral VA aplasia in three countries, USA, Japan, and India, were discovered.
2. Two-thirds of VA aplasia cases belonged to patients of ages 31 to 80.
3. Although there was no significant sex difference in appearance of VA aplasia, male gender was more common in cases of bilateral VA aplasia.
4. The side of the VA aplasia has significant influence on the side of CVBA persistence, or vice versa.
5. CVBA persistence is significantly more common on the left side in cases of uni- and bilateral VA aplasia.
6. Associated aplasia of other arteries was more common in cases of unilateral VA aplasia.
7. The left VA was more commonly hypoplastic in cases of single right VA aplasia than the right VA in cases of single left VA aplasia.
8. There was high incidence of cerebral artery stenosis, occlusion, cerebral infarction, or stroke in cases of single left and bilateral VA aplasia.
9. Aneurysms of definitive arteries were more frequent in cases of single right VA aplasia than in cases of single left VA aplasia.

## Figures and Tables

**Table 1:** Distribution of single cases of the vertebral artery (VA) aplasia in various countries.

Side of VA aplasia	Countries^*∗*^ [references]																								∑
Bilaterally				Canada [14, 25, 38]	China [15, 34]		France [33, 35]	Germany [20]			India [16, 21, 26, 30, 39]	Israel [22]	Italy [23, 31, 40]	Japan [17, 27, 29, 36]	Lithuania [44]				Spain [41]			Turkey [28]	UK [42]	USA [18, 19, 24, 32, 37, 43]	

Number				3	2		2	1			5	1	3	4	1				1			1	1	6	**31**

Left	Australia [64]	Belgium [74]			China [45, 81]	Croatia [65]	France [75]	Germany [55]	Greece [76]		India [30, 46]		Italy [47, 66]	Japan [48–52, 61–63, 67]				South Korea [57]	Spain [53, 56]	Sweden [54]	Switzerland [58]	Turkey [59, 68, 80, 109]		USA [60, 69–73, 77–79]	

Number	1	1			2	1	1	1	1		3		2	9				1	7	1	1	4		10	**46**

Right			Brazil [95]	Canada [96]	China [108, 110]		France [75, 93, 97]	Germany [90]	Greece [98]	Grenada [82]	India [86, 106]		Italy [40]	Japan [83, 84, 87, 94, 99, 100]		Serbia^*∗∗*^ [101]	South Africa [88]	South Korea [85, 91]				Turkey [92, 107]		USA [89, 102–105]	

Number			1	1	2		3	1	1	1	2		1	6		2	1	2				2		5	**31**

∑	**1**	**1**	**1**	**4**	**6**	**1**	**6**	**3**	**2**	**1**	**10**	**1**	**6**	**19**	**1**	**2**	**1**	**3**	**8**	**1**	**1**	**7**	**1**	**21**	**108**

^*∗*^Alphabetical order. ^*∗∗*^Recent case is included.

**Table 2:** Distribution of the vertebral artery (VA) aplasia according to the gender.

Side of VA aplasia	Female	Male	Unknown
	Number (%)		
Bilateral	11/108 (10.18)	19/108 (17.59)	1/108 (0.92)
Left	24/108 (22.22)	20/108 (18.52)	2/108 (1.85)
Right	14/108 (12.96)	14/108 (12.96)	3/108 (2.77)

Total	49/108 (45.37)	53/108 (49.07)	6 (5.55)

**Table 3:** Distribution of 108 cases of the vertebral artery (VA) aplasia according to age.

Age of patients	Number of cases of VA aplasia			
	Bilaterally	Left side	Right side	(∑ = 108)
Stillborn		1		1
Newborn (few hours after birth)		1		1
Neonate (≤28 days)	1	1		2
Suckling (≤12 months)	1	5		6
Tot (≤3 years)		3		3
Preschool age (≤5 years)			1	1
School age (6–12)	1			1
Adolescent (13–18)		1		1
19–30	1	2	1	4
31–40	2	3	8	13
41–50	4	4	4	12
51–60	6	9	3	18
61–70	11	7	5	23
71–80	3	5	6	14
>81		2		2
Unknown age	1	2	3	6

**Table 4:** Sixteen (16/46) literature cases^a^ of total aplasia of the left vertebral artery (VA) and hypoplasia of the right VA.

Number	Gender/age [reference]	Initial symptoms or reasons of research	Left VA aplasia			
			Associated vascular aplasia	Associated persistent CVBA and/or other vascular abnormalities		
			Other arteries and/or veins	Type of persistent CVBA (vascular source)	Other variants and/or abnormalities	Diagnosed pathology
(1)	F/0 [77]	Stillborn		Left PPHA (ICA)	Hypoplastics right VA/left A1 and PCoA	

(2)	M/14 [47]	Headache/nausea/slight neck stiffness	Left PCoA	Left PPIA (ICA)	Hypoplastic right VA terminated as the PICA Low left CCA bifurcation Accessory right MCA	SAH

(3)	M/28 [55]	Cavernous hemangioma in the skin of the forehead		Left PPHA (ICA)	Hypoplastic right VA Dilatation of Galen's vein	

(4)	F/43 [45]	Headache		Left PPIA (ICA)	Hypoplastic right VA	Aneurysm of the left PICA SAH/IVH

(5)	M/51 [76]	Loss of consciousness		Left PPHA (ICA)	Hypoplastic right VA	

(6)	M/52 [75]	Right hemiparesis/aphasia	Left AICA and PCoA	Left PPHA (ICA)	Hypoplastic right VA Left PICA and ASA of PPHA origin Ectatic left CCA Dolicho-left ICA BT-left CCA common trunk	

(7)	F/54 [52]	Headache/weakness of the right lower limb	Bilateral PCoAs	Left PPHA (ICA)	Hypoplastic right VA	Aneurysms of the right ACA and PPHA–BA junction

(8)	F/55 [68]	Coma		Left PPIA (ICA)	Hypoplastic right VA	Calcified atheromatous plaques in both carotid systems Lesions from the level of the mesencephalon to the both thalami

(9)	F/55 [78]	Self-audible left neck bruit		Left PPHA (CCA)	Hypoplastic right VA	Stenosis of the left CCA, ICA, and PPHA

(10)	M/61 [65]	Speech disorder/left supranuclear facial palsy	Right ACA	Left PPIA (ICA)	Hypoplastic right VA	Calcified atheromatous plaques in both carotid systems and PPIA. Stenosis of bilateral SAs Aneurysm of the right SA. Lacunar ischemic changes in basal ganglia

(11)	F/62 [60]	Intermittent diplopia	Left PCoA	Left PPHA (ICA)	Hypoplastic right VA. BT and left CCA common origin	Irregular lesion of the left CCA bifurcation and a moderate stenosis with ulceration of the proximal ICA

(12)	F/63 [70]	Transient right hand weakness/left amaurosis fugax		Left PPIA (ECA)	Hypoplastic right VA terminated as the PICA	Severe ICA stenosis

(13)	F/65 [79]	Left carotid bruit	Both PCoAs	Left PPHA (ICA)	Hypoplastic right VA terminated as the PICA	Stenosis of the left PPHA origin

(14)	F/73 [74]	Acute paresis in the left arm	Left PCoA	Left PPHA (ICA)	Hypoplastic right VA	Bleeding in the right parietooccipital lobe

(15)	F/78 [66]	Transischemic attacks with right-sided paresis		Left PPIA (ICA)	Hypoplastic right VA did not form the BA	Stenosis of the left CCA bifurcation Ulcerated plaque extended into the ECA, ICA, and PPIA origin

(16)	M/83 [63]	Cerebral infarction	Bilateral PCoAs	Left PPIA (ECA) Lateral type of the left PPTA (ICA–C3 part)	Hypoplastic right VA supplied only ipsilateral PICA Distal branch of the left OA of PPIA origin	

^a^Cases according to age are listed; number “0” for stillborn status is used; other Arabian numbers indicate age in years; CVBA, carotid-vertebrobasilar anastomosis; F, female; PPHA, persistent primitive hypoglossal artery; ICA, internal carotid artery; A1, precommunicating part of the anterior cerebral artery; PCoA, posterior communicating artery; M, male; PPIA, persistent proatlantal intersegmental artery (independently of its subtype); PICA, posterior inferior cerebellar artery; CCA, common carotid artery; MCA, middle cerebral artery; SAH, subarachnoid hemorrhage; IVH, intraventricular hemorrhage; AICA, anterior inferior cerebellar artery; ASA, anterior spinal artery; BT, brachiocephalic trunk; ACA, anterior cerebral artery; BA, basilar artery; SA, subclavian artery; ECA, external carotid artery; PPTA, persistent primitive trigeminal artery; C3, cavernous part of the internal carotid artery; OA, occipital artery.

**Table 5:** Thirteen (13/31) literature cases^a^ of total aplasia of the right vertebral artery (VA) and hypoplasia of the left VA.

Number	Gender/age (author)	Initial symptoms or reasons of research	Right VA aplasia			
			Associated vascular aplasia	Associated persistent CVBA and other variants and/or abnormalities		
			Other arteries and/or veins	Type of persistent CVBA (vascular source)	Other variants and/or abnormalities	Diagnosed pathology
(1)	F/30 [84]	Headache (of the 37th-week pregnant woman)		Right PPHA (ICA)	Hypoplastics left ICA and VA AVM	Stenosis of the left ICA at the entrance from the carotid siphon Intracranial hemorrhage

(2)	F/31 [97]	History of pain beginning in the right temporomandibular joint		Right PPHA (ICA)	Hypoplastic left VA Displaced right temporal lobe	Chondroblastoma

(3)	F/34 [85]	Headache		Right PPIA^*∗*^ (ICA)	Hypoplastic left VA	Ruptured aneurysm of the left AChA Unruptured aneurysm of the right AChA

(4)	M/37 [88]	Left-sided weakness	Bilateral PCoA Right ACA	Unusual right CBA (ICA)	Hypoplastic left VA Right CBA coursing through the jugular foramen and distributed right PICA Aberrant right SA	

(5)	M/41 [92]	Vertigo	Bilateral ECA	Right PPIA (ICA)	Hypoplastic left VA Left CCA-SA common trunk Bilaterally CCA distributes ECA branches	

(6)	F/43 [99]	SAH	Bilateral PCoA	Right PPHA	Hypoplastic left VA Kinking of the right P1	Aneurysm of the BA bifurcation Partial occlusion of the right P1

(7)	F/49 [103]	SAH		Right PPHA (ECA)	Hypoplastic left VA	Right PICA aneurysm

(8)	M/58 [93]	Right carotid bruit	Right PCoA	Right PPIA (ICA)	Hypoplastics left VA and PCoA	Tight stenosis of the right ICA origin

(9)	M/62 [87]	Vertigo/left upper extremity weakness	Bilateral PCoA	Right PPHA (ICA)	Hypoplastic left VA	Stenosis of the right ICA

(10)	M/66 [95]	Sudden visual blurring		Right PPHA (ICA)	Hypoplastic left VA. Right SA, left VA, and both CCAs originated directly from the aortic arch	Occlusion of bilateral PCA. Stenosis of the right CCA and ICA and left MCA. Right occipital ischemic stroke

(11)	F/74 [96]	Bilateral carotid bruits	Right PCoA	Right PPHA (ICA)	Hypoplastic left VA	Occlusion of the left ICA. Stenosis of the left ECA/right ICA/ECA

(12)	F/74 [94]	Loss of consciousness		Right PPIA (ECA)	Hypoplastic left VA terminated as the PICA	SAH. Aneurysm of the MCA trifurcation

(13)	(U) [98]	Anatomy dissection		Right PPHA (ICA)	Hypoplastic left VA	

^a^Cases according to the age are listed; Arabian numbers indicate age in years; CVBA, carotid-vertebrobasilar anastomosis; F, female; PPHA, persistent primitive hypoglossal artery; ICA, internal carotid artery; AVM, arteriovenous malformation; PPIA^*∗*^, persistent proatlantal intersegmental artery (independently of its type); AChA, anterior choroidal artery; M, male; PCoA, posterior communicating artery; ACA, anterior cerebral artery; CBA, carotid-basilar anastomosis; PICA, posterior inferior cerebellar artery; SA, subclavian artery; ECA, external carotid artery; CCA, common carotid artery; SAH, subarachnoid hemorrhage; P1, precommunicating part of the posterior cerebral artery; BA, basilar artery; PCA, posterior cerebral artery; MCA, middle cerebral artery; U, unknown gender.

**Table 6:** Left VA aplasia versus bilateral VA aplasia.

Number	Parameters			Left VA		Bilateral VA	
				46 cases		31 cases	
(1)	Incidence			59.74%		40.26%	

(2)	Gender						
	Female			31.16%		14.28%	
	Male			25.97%		24.67%	
	Female/male/unknown gender			45.44%/50.64%/3.89%			

(3)	Persistence of CVBA						
	Unilateral persistence of CVBA			82.60%		59.07%	
	Bilateral persistence of CVBA			4.35%		34.48%	
	Persistence of two different CVBAs			4.34%		3.22%	
	Persistence of determined CVBA						
	PPIA			43.47%		54.83%	
	PPHA			34.78%		32.25%	
	PPTA			13.04%		9.67%	
	Unusual arterial anastomoses					6.45%	

(4)	Additional vascular variants						
		*Uni-*	*Bi-*	*Uni-*	*Bi-*	*Uni-*	*Bi-*
		CCA		3.22%			
		ICA		6.52%			
		ACA		3.22%			
	Associated aplasia of other blood vessels	PCoA	PCoAs	8.69%	8.69%	3.22%	9.67%
		BA				3.22%	
		AICA		3.22%			
		SA		3.22%			
		Some dural sinuses/bilateral IJV				3.22%	
	Hypoplastic right VA			34.78%			
	Unusual origin or side branches or termination or hypoplasia of other arteries or additional anastomoses			39.13%		41.93%	

(5)	Associated vascular pathology						
	Aneurysms of definitive arteries			8.69%		19.35%	
	Aneurysms of CVBAs			4.34%		3.22%	
	Different cerebral pathology (except that of aneurysms)			41.30%		41.93%	
	Noncerebral pathology			4.34%		0	

VA, vertebral artery; CVBA, carotid-vertebrobasilar anastomosis; PPIA, persistent primitive proatlantal intersegmental artery (without mark of the type); PPHA, persistent primitive hypoglossal artery; PPTA, persistent primitive trigeminal artery; ECA, external carotid artery; ICA, internal carotid artery; ACA, anterior cerebral artery; PCoA, posterior communicating artery; BA, basilar artery; SA, subclavian artery; IJV, internal jugular vein.

**Table 7:** Single right VA aplasia versus bilateral VA aplasia.

Number	Parameters			Right VA		Bilateral VA	
				31 cases		31 cases	
(1)	Incidence			40.32%		59.68%	

(2)	Gender						
	Female			22.58%		17.74%	
	Male			22.58%		30.64%	
	Female/male/unknown gender			40.32%/53.12%/6.45%			

(3)	Persistence of CVBA						
	Unilateral persistence of CVBA			80.64%		59.07%	
	Bilateral persistence of CVBA			0		34.48%	
	Persistence of two different CVBAs			0		3.22%	
	Persistence of determined CVBA						
	PPIA			16.13%		54.83%	
	PPHA			51.61%		32.25%	
	PPTA			6.45%		9.67%	
	Unusual arterial anastomoses			6.45%		6.45%	

(4)	Additional vascular variants						
	Associated aplasia of other vessels	*Uni-*	*Bi-*	*Uni-*	*Bi-*	*Uni-*	*Bi-*
			ECAs		3.22%		
		ICA		6.45%			
		ACA		3.22%			
		PCoA	PCoAs	12.90%	6.45%	3.22%	9.67%
		BA		3.22%		3.22%	
		SA branches		3.22%			
	Hypoplastic left VA			41.93%			
	Unusual origin or side branches or termination or hypoplasia of other arteries or additional anastomoses			32.25%		41.93%	

(5)	Associated vascular pathology						
	Aneurysms of definitive arteries			25.80%		16.13%	
	Aneurysms of CVBAs			3.22%		3.22%	
	Different cerebral pathology (except that of cerebral aneurysms)			35.48%		35.48%	
	Noncerebral pathology			9.67%		3.22%	

VA, vertebral artery; CVBA, carotid-vertebrobasilar anastomosis; PPIA, persistent primitive proatlantal intersegmental artery; PPHA, persistent primitive hypoglossal artery; PPTA, persistent primitive trigeminal artery; CCA, common carotid artery; ICA, internal carotid artery; ACA, anterior cerebral artery; PCoA, posterior communicating artery; BA, basilar artery; AICA, anterior inferior cerebellar artery; SA, subclavian artery.

**Table 8:** Single left VA aplasia versus right VA aplasia.

Number	Parameters			Left VA aplasia		Right VA aplasia	
				46 cases		31 cases	
(1)	Incidence			59.74%		40.26%	

(2)	Gender						
	Female			31.16%		18.18%	
	Male			25.97%		18.18%	
	Female/male/unknown gender			49.34%/44.15%/6.49%			

(3)	Persistence of CVBA						
	Unilateral persistence of CVBA			82.60%		80.64%	
	Bilateral persistence of CVBA			4.34%		0	
	Persistence of two different CVBA			4.34%		0	
	Persistence of determined CVBA						
	PPIA			43.47%		16.13%	
	PPHA			34.78%		51.61%	
	PPTA			13.04%		6.45%	
	Unusual arterial anastomoses			0		6.45%	

(4)	Additional vascular variants						
	Associated aplasia of other blood vessels	*Uni-*	*Bi-*	*Uni-*	*Bi-*	*Uni-*	*Bi-*
		CCA		3.22%		3.22%	
		ECA					3.22%
		ICA		6.45%		6.45%	
		ACA		3.22%		3.22%	
		PCoA	PCoAs	8.69%		12.90%	6.45%
		BA				3.22%	
		AICA		3.22%			
		SA trunk		3.22%			
		SA branches				3.22%	
	Hypoplastic (opposite) VA			34.78%		41.93%	
	Unusual origin or side branches or termination or additional anastomoses			39.13%		32.25%	

(5)	Associated vascular pathology						
	Aneurysms of definitive arteries			8.69%		25.80%	
	Aneurysms of CVBAs			4.34%		3.22%	
	Other cerebral pathology			41.30%		35.48%	
	Noncerebral pathology			4.34%		9.67%	

VA, vertebral artery; CVBA, carotid-vertebrobasilar anastomosis; PPIA, persistent primitive proatlantal intersegmental artery; PPHA, persistent primitive hypoglossal artery; PPTA, persistent primitive trigeminal artery; CCA, common carotid artery; ECA, external carotid artery; ICA, internal carotid artery; ACA, anterior cerebral artery; PCoA, posterior communicating artery; BA, basilar artery; SA, subclavian artery.
