# Erratum to “Unilateral Aplasia versus Bilateral Aplasia of the Vertebral Artery: A Review of Associated Abnormalities”

**DOI:** 10.1155/2018/8638434

**Published:** 2018-02-21

**Authors:** L. Vasović, M. Trandafilović, S. Vlajković, G. Djordjević, M. Daković-Bjelaković, M. Pavlović

**Affiliations:** ^1^Department of Anatomy, Faculty of Medicine, University of Niš, 81 Blvd. Dr. Zoran Djindjić, 18000 Niš, Serbia; ^2^Health Center Niš, 15 Vojvode Tankosića St., 18000 Niš, Serbia

In the article titled “Unilateral Aplasia versus Bilateral Aplasia of the Vertebral Artery: A Review of Associated Abnormalities” [1], there was an error in reference [80], which should be corrected as follows.

O. Yeniceri, N. Cullu, M. Deveer and R. M. Kilinc, “Persistent trigeminal artery anomaly with concomitant basilar artery hypoplasia,”* Austin Journal of Radiology*, vol. 2, no. 2, p. 1014, 2015.
